# Mesopredator Release by an Emergent Superpredator: A Natural Experiment of Predation in a Three Level Guild

**DOI:** 10.1371/journal.pone.0015229

**Published:** 2010-12-06

**Authors:** Nayden Chakarov, Oliver Krüger

**Affiliations:** Department of Animal Behaviour, University of Bielefeld, Bielefeld, Germany; Institute of Ecology, Germany

## Abstract

**Background:**

Intraguild predation (IGP) is widespread but it is often neglected that guilds commonly include many layers of dominance within. This could obscure the effects of IGP making unclear whether the intermediate or the bottom mesopredator will bear higher costs from the emergence of a new top predator.

**Methodology/Principal Findings:**

In one of the most extensive datasets of avian IGP, we analyse the impact of recolonization of a superpredator, the eagle owl *Bubo bubo* on breeding success, territorial dynamics and population densities of two mesopredators, the northern goshawk *Accipiter gentilis* and its IG prey, the common buzzard *Buteo buteo*. The data covers more than two decades and encompass three adjacent plots. Eagle owls only recolonized the central plot during the second decade, thereby providing a natural experiment. Both species showed a decrease in standardized reproductive success and an increase in brood failure within 1.5 km of the superpredator. During the second decade, territory dynamics of goshawks was significantly higher in the central plot compared to both other plots. No such pattern existed in buzzards. Goshawk density in the second decade decreased in the central plot, while it increased in both other plots. Buzzard density in the second decade rapidly increased in the north, remained unchanged in the south and increased moderately in the center in a probable case of mesopredator release.

**Conclusions/Significance:**

Our study finds support for top-down control on the intermediate mesopredator and both top-down and bottom-up control of the bottom mesopredator. In the face of considerable costs of IGP, both species probably compete to breed in predator-free refugia, which get mostly occupied by the dominant raptor. Therefore for mesopredators the outcome of IGP might depend directly on the number of dominance levels which supersede them.

## Introduction

Intraguild predation (IGP) is the complex interaction between member species of a guild, that both compete for resources and kill each other [1]. IG predators can actively persecute competitors so that IG prey experiences an even greater risk of attack than extraguild prey [2]. However, they are potentially less adapted to negate predation through strategic and tactical responses. In predatory guilds, IG prey i.e. mesopredators are often limited by predation but resilient towards other stress factors. Hence, when the larger apex predator is removed, the smaller IG prey can proliferate in a phenomenon known as mesopredator release, normally with significant consequences for the underlying food web [2]. Coexistence of apex predators and mesopredators can depend on ecosystem productivity and prey abundance [3], but also on habitat complexity [4]. The latter is important for prey refugia but probably also for competitors limited to a greater degree by habitat-sharing than by food competition. While apex predators often become extinct under anthropogenic influence, delivering many examples of mesopredator releases [2], [5], only few studies have shown a restoration of the mesopredator suppressed state after reintroduction or recolonization of the apex predator [3], [6], [7], [8].

Most studies of IGP observe the influence of a top predator on a mesopredator. Yet guilds are usually more complex, having more than two dominance/trophic levels within. Depending on the relative strength of both predation and competition between the involved guild members, the effects of IGP can be expected to cascade down the dominance hierarchy within the guild or not [9], [10], [11], [12]. Examples of either are scarce. Two of the possible outcomes for a simple three-level guild are: 1. Under IGP pressure the intermediate guild member deflects by exploiting recourses otherwise used by species positioned lower in the guild hierarchy. The emergence of a top predator leads to more severe restriction of the bottom mesopredator. 2. The emergent top predator restricts the intermediate mesopredator so that the bottom guild member actually experiences a decrease in IGP pressure. Thus even a slight complication of guild structure could alter the total biomass and predatory capacity of the involved species in not well examined ways, with repercussions for the underlying community and ecosystem biodiversity [13].

In the present study we analyse one of the most extensive datasets on avian IGP, making use of a natural experiment of superpredator treatment [14]. We examine how the recolonization of an apex predator, the eagle owl *Bubo bubo*, affects reproductive success and population density of two mesopredators, the northern goshawk *Accipiter gentilis* and the common buzzard *Buteo buteo*, the former also being an IG predator for the latter. We predict that: 1. Apex predator proximity will lead to a decrease in breeding success of both mesopredators. 2. The dominant mesopredator will spatially avoid the apex predator, leading to higher territory dynamics in the area with apex predators. 3. The inferior mesopredator, having a higher population density will not be able to spatially avoid the apex predator without falling under extreme inter- and intraspecific competition. Thus no detectable differences in territory dynamics in the area with apex predators should occur. 4. Population density of both mesopredators will decrease in the area inhabited by apex predators. However because of their different trophic/dominance position one will probably bear more damage than the other.

## Results

### Reproductive success of goshawks

**Figure 1:**
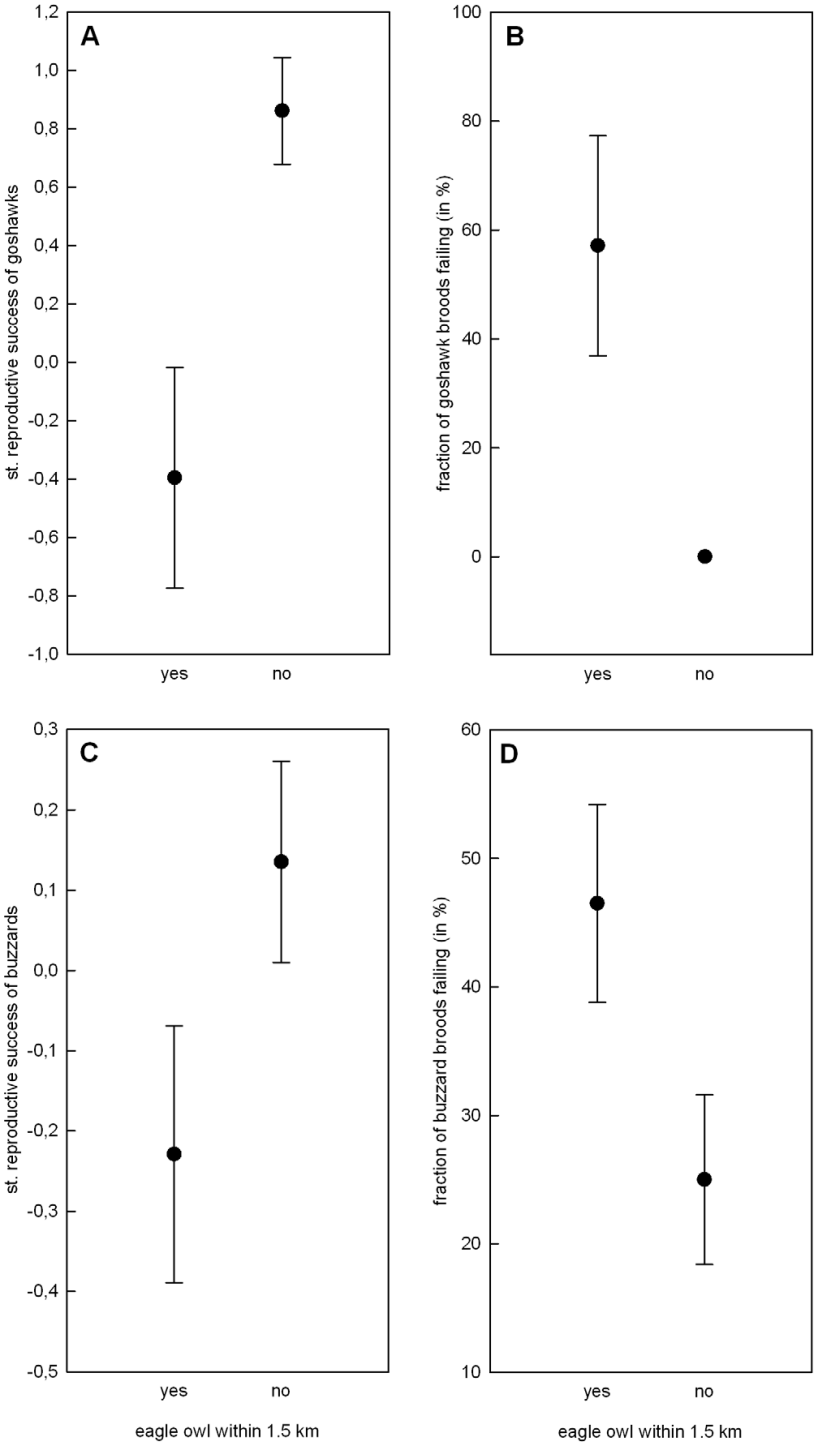
Eagle owl influence on reproductive success of goshawks and buzzards. Standardized reproduction rate and fraction of nests failing (± SE) of goshawks A, B and common buzzards C, D breeding outside or within 1.5 km of an eagle owl nest within the central plot.

Reproductive success of goshawks for the whole study period was predicted by plot of breeding, period of eagle owl establishment and the interaction between both (plot χ^2^ = 19.48, p<0.001, period χ^2^ = 11.06, p = 0.011, plot × period χ^2^ = 10.99, p = 0.004, model weight = 0.52). After eagle owl recolonization, the only significant predictor of reproductive success was plot of breeding (plot χ^2^ = 17.12, p<0.001, model weight = 0.99). For that period in the central plot, the only predictor of goshawk reproductive success was the presence of an eagle owl breeding pair within 1.5 km (Figure 1A, eagle owl within 1.5 km χ^2^ = 9.91, p = 0.002, model weight = 0.87).

The best model explaining goshawk brood failure over the whole study period contained plot, period of eagle owl establishment, nearest neighbour distance (NND) and the three two-way interactions between them (plot χ^2^ = 22.71, p<0.001, period χ^2^ = 10.63, p = 0.031, NND χ^2^ = 14.83, p = 0.005, plot × NND χ^2^ = 11.63, p = 0.003, plot × period χ^2^ = 7.15, p = 0.028, plot × NND χ^2^ = 13.81, p = 0.001, period × NND χ^2^ = 2.14, p = 0.14, model weigh  = 0.278). The next best model with slightly lower model weight (0.276) did not include the interaction period × NND. After eagle owls establishment, the best explanatory model of brood failure consisted of plot, NND and their interaction (plot χ^2^ = 19.81, p<0.001, NND χ^2^ = 10.73, p = 0.013, plot × NND χ^2^ = 10.44, p = 0.005, model weight  = 0.43). Then in the central plot, brood failure was predicted by the presence of an eagle owl breeding pair within 1.5 km and marginally by goshawk NND (Figure 1B, eagle owl within 1.5 km χ^2^ = 7.22, p = 0.007, NND χ^2^ = 3.31, p = 0.069, model weight  = 0.51).

### Reproductive success of buzzards

Over the entire study period, the only significant predictors of reproductive success were the morphs of both breeding partners (female morph χ^2^ = 21.41, p<0.001, male morph χ^2^ = 26.91, p<0.001, model weight  = 0.83). After eagle owl recolonization, reproductive success was best predicted by the random term of female identity alone (model weight  = 0.50). The second best model included solely plot of breeding (plot χ^2^ = 7.64, p = 0.022, model weight  = 0.41). Similarly within the central plot, reproductive success was best predicted by the random term of female identity only (model weight  = 0.225). The second best model contained the marginally significant occurrence of an eagle owl breeding pair within 1.5 km of the buzzard nest (Figure 1C, eagle owl within 1.5 km χ^2^ = 3.23, p = 0.071, model weight  = 0.189).

Buzzard brood failure for the period 1989–2009 was predicted by period of eagle owl establishment, male and female morph, vole score of the year, NND and the interaction between eagle owl period and vole score (period χ^2^ = 35.69, p<0.001, vole score χ^2^ = 72.46, p<0.001, female morph χ^2^ = 13.11, p = 0.001, male morph χ^2^ = 8.26, p = 0.017, NND χ^2^ = 6.473, p = 0.011, period × vole score χ^2^ = 25.14, p<0.001, model weight  = 0.37). After eagle owl establishment, brood failure in buzzards was most constantly predicted by vole score and plot of breeding (vole score χ^2^ = 59.02, p<0.001, plot χ^2^ = 7.99, p = 0.018, model weight  = 0.136). Next best models included NND and distance to goshawk. For the same period in the central plot, the best model of brood failure consisted of the occurrence of an eagle owl within 1.5 km (Figure 1D, χ^2^ = 4.43, p = 0.035, model weight  = 0.231). Inferior models included male and female morph and vole score, which remained not significant predictors.

### Territory dynamics

**Figure 2:**
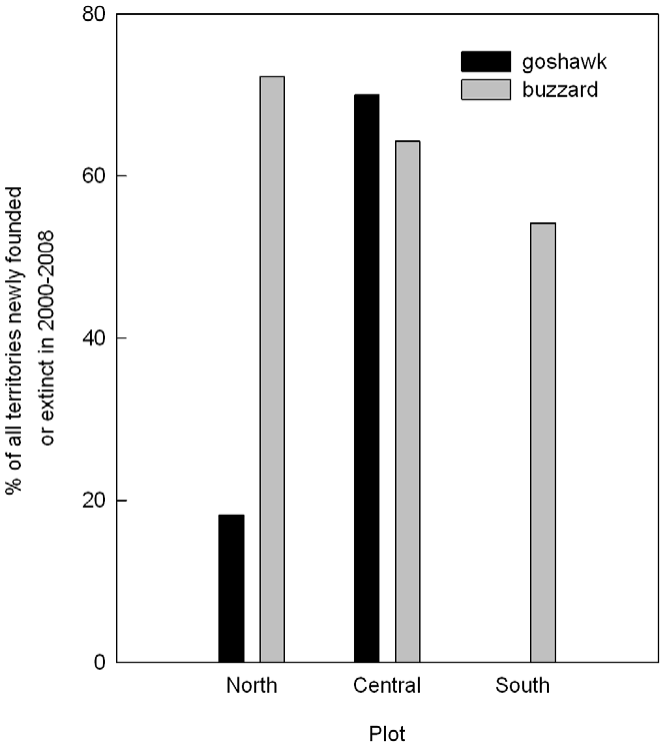
Eagle owl influence on territory dynamics of goshawks and buzzards. Territory dynamics, measured as the joint percentage of all territories that were newly founded or became extinct in the period 2000–2009 when eagle owls colonized the central plot. Percentages were significantly different in goshawks (black bars), but not buzzards (grey bars).

For goshawks, we found a significantly different territory dynamics between the three plots measured in newly founded or extinct territories after the area was colonized by eagle owls (Figure 2, χ^2^ = 8.74, p = 0.013). This was mainly due to new territories getting established in the central plot after 2000 (χ^2^ = 8.51, p = 0.014), rather than extinctions (χ^2^ = 3.18, p = 0.204). Buzzards did not show different territory dynamics between both decades and the three plots of study. There were neither more newly founded territories (χ^2^ = 3.72, p = 0.156) nor extinct ones after 2000 in any of the three plots (χ^2^ = 1.51, p = 0.470).

### Population density

**Figure 3:**
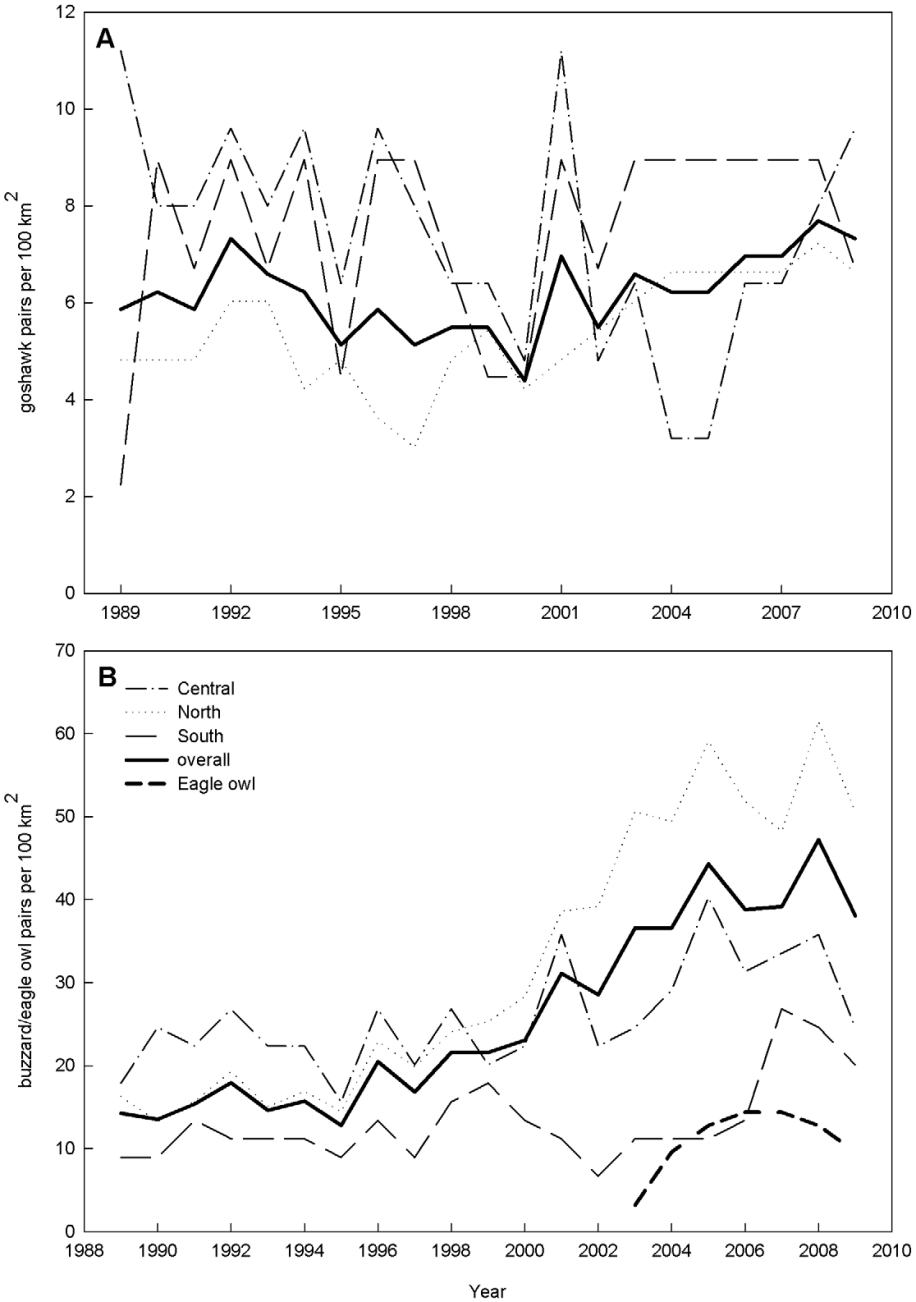
Population dynamics of goshawks, buzzards and eagle owls. Dynamics of goshawk A and buzzard B and eagle owl population densities in the period 1989–2009 in three adjacent plots in Eastern Westphalia, Germany. Eagle owls inhabit the central plot only and densities are calculated over its surface.

**Figure 4:**
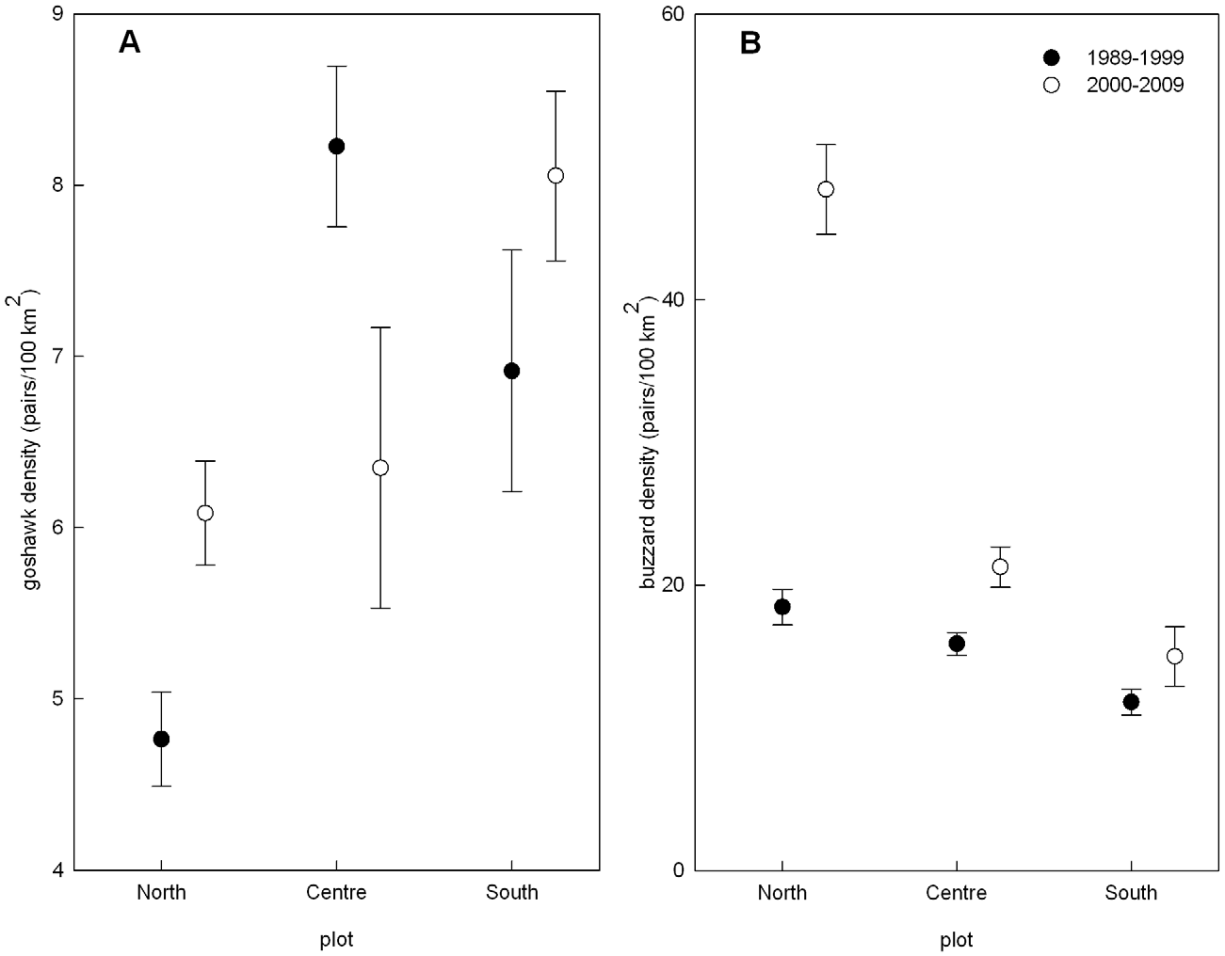
Goshawk and buzzard densities in the three plots and two decades of study. Densities (± SE) of goshawks A and buzzards B in the study area in Eastern Westpahlia in relation to the decade of study (black: 1989–1999, white: decade of eagle owl recolonization, 2000–2009) and plot of study.

Goshawk density was best explained by plot and its interaction with decade (Figure 3A and 4A; plot χ^2^ = 18.0127, p<0.001; plot × decade χ^2^ = 10.81, p = 0.004; decade χ^2^ = 0.1899, p = 0.663; model weight  = 0.42).

Buzzard density was best explained by plot, goshawk density, their interaction and decade (Figure 3B and 4B; plot χ^2^ = 172.84, p<0.001; goshawk density χ^2^ = 29.06, p<0.001; goshawk density × plot χ^2^ = 8.48, p = 0.014; decade χ^2^ = 54.30, p<0.001; model weight  = 0.45).

## Discussion

Congruent with our predictions, there were different patterns of change in population density and territory occupation dynamics of goshawks and buzzards while reproductive success of both species decreased when part of the study area became recolonized by the eagle owl superpredator. As expected, population density of goshawks in the central area dropped between 2000 and 2009 which was by then under high eagle owl influence, while it increased in both other areas. This is exactly what should be expected of mesopredators under strong top-down control [3]. At the same time, however, buzzard density somewhat surprisingly slightly increased in the area colonized by eagle owls. This differs from the southern plot, where the change was not significant. It also markedly differs to what happened in the northern part of the population, where density rapidly increased. On the one hand buzzards in the central plot probably experienced a second degree mesopredator release from goshawk pressure through eagle owl predation on goshawks. Similar top-down release within a guild has been found in owl assemblages when pressure from tawny owls decreased through eagle owl predation [11]. While a top-down control of buzzard density by goshawks seems probable, it could have a stronger competition than predation element [15]. Otherwise buzzards would have to be more severely influenced by goshawk than by eagle owl presence. However our analyses of buzzard breeding performance showed that if there was any effect by an IG predator at all, it was only by eagle owl and not by goshawk proximity. Another possible mechanism of increase in density under superpredator influence would be a source-sink dynamic, if the northern subpopulation fuels the central and the south with young individuals, founding new territories and substituting deceased and killed territory holders [16]. Our data does not yet allow us to examine this possibility because not enough individually marked buzzards have recruited in the population yet. Most probably buzzards are also under bottom-up control reinforced by some differing habitat features of the three areas. The northern area is very close to the optimal buzzard habitat with small woodlots separated by agricultural plots. Additionally, the loamy soils in the north sustain higher yields and vole abundances, permitting the increase in buzzard densities. Meanwhile in the central ridge and in the south, the forest patches are much larger and soils are sandy and not as fertile in the south. Soil fertility is positively related to plant biomass and prey abundance [3], hence conditions are presumably not as good in the south compared to the center and north. Territory size in buzzards is between 1 and 3 km^2^ [17] so they predominantly hunt in the vicinity of their nest site. Jointly, these factors might prohibit a great increase in buzzard density in the center or south. Low productivity environments are expected to facilitate predator coexistence if prey is not sufficient to support high superpredator densities [2]. However eagle owl densities in our central plot were relatively high (up to 12 breeding pairs per 100 km^2^) pointing to food competition not being the most influential aspect in this IGP system. Additionally, according to the findings of Elmhagen et al. [3], precisely under such barren conditions should one expect strong top-down control on buzzard density. While this might have occurred it probably has been compensated and outweighed by the release from goshawk pressure. Similarly, other studies of IGP have also found a complex interaction of top-down and bottom-up control on the densities of the involved populations [2], [3]. Although we made use of a natural experiment, our study is not able to distinguish clearly between habitat and cascade effects.

Eagle owls concentrate their hunting efforts within 2–3 km of the nest [11] and most buzzard nests in the central part and some of those in the southern part of our study area are within that range of an eagle owl nest. If buzzards in both of these plots fall under high eagle owl influence, this could help to explain why there is only a slight difference in buzzard density development between center and south and would promote the explanation of a top-down effect of eagle owls on buzzard density. In such a case the same pattern probably should have been found for goshawks as well. However, as eagle owls locate their prey through its displays and nest conspicuousness [18], goshawks could have an advantage because they are more cryptic and breed in greater forest patches than buzzards; goshawk nests could be harder to find at greater distances from the eagle owl nest. Moreover, fear of predation could play a smaller role in goshawks, as they seem to be sensitive to different stress types than buzzards [15]. While such fear tolerance might exist at intermediate predation risk for the southern plot, this probably is not the case for the small distances from eagle owls within the central plot. A similar abrupt change in predator-avoidance tactics has been found in tawny owls which are distance sensitive at intermediate eagle owl densities but avoid risky habitats at high eagle owl densities [11].

Territory dynamics of goshawks was significantly higher in the central plot, inhabited by eagle owls. This is in line with our prediction. This pattern could point to a typical ecological trap where inexperienced goshawks found territories near eagle owls and get predated relatively swiftly [19]. However, since the main difference in territory dynamics was in territory establishments, most goshawks probably manage to withdraw to refugia from eagle owls within the central plot. Buzzards, on the other hand, showed insignificant differences in territory dynamics between plots. Thus their turnover rates were not influenced by the presence of eagle owls. Most buzzards might have no access to such refugia because of potential scarcity and occupation by goshawks. In line with this reasoning, the majority of newly founded goshawk territories in the central plot were former buzzard territories. The density of buzzards in the area and the suboptimal habitat could additionally exert a higher pressure on keeping the focal territory. Both effects probably work in conjunction as an additional element of the documented competition between goshawks and buzzards for optimal nest sites [15]. Also buzzards always have a goshawk nearby, posing as an IG predator. So the emergence of another predator such as the eagle owl might be no additional fear-inducing factor that would justify territory desertion. Such habituation transfer has been found in squirrel escape response to human, coyote and hawk threats but it remains questionable whether it could take place between ambush predators such as eagle owls and goshawks [20]. It is hard to define the key features of potential refugia from eagle owl influence. The main one probably is direct distance from the eagle owl nest [11], [16]. This is supported by the negative effects on goshawk and buzzard reproduction, which we found within 1.5 km of eagle owl nests. This is similar to the findings of Sergio et al. [16] where the negative effect of eagle owls on black kite reproduction was mainly for nests closer than 1.5 km and no successful breeding attempt took place within 1 km of an eagle owl nest. Even though in our case this was not as severe, within the same habitat, goshawk and buzzard nests closer than 1.5 km seemed to underperform, while nests, which were further away, overperformed. This was mainly due to a two-fold increase in nest failure rates. In order to stay within refugia goshawks probably have to abandon parts of the central ridge although it contains the biggest patches of wood in the study area and many former prime nesting sites [21]. Since many areas in central Europe have recently been or currently get recolonized by eagle owls, mesopredators positioned sufficiently low in the dominance hierarchy could experience a significant benefit from this process, even though in the case of common buzzards this is only one part of a success story.

While understanding of IGP dynamics is rapidly growing it is important that guilds are complex and usually many species are involved. Only few studies of vertebrate guilds have managed to recognize the implications of this added complexity. Our study suggests that the impact of a superpredator on a mesopredator could depend on whether there is an additional mesopredator between them or not. Depending on the strength of top-down, competition and bottom-up processes, each new species could trigger cascading effects on other species in an unpredictable way [22]. This could have important consequences for wildlife management because it can make the impact of mesopredators on underlying communities less predictable [22] and alter the association of apex predators and high biodiversity [13].

## Materials and Methods

### Ethics statement

No permits were required since the study area did not contain any strict protected areas and due to the observational nature of the data collection.

### Study species

The common buzzard is a medium sized raptor (♂ 525–1183 g, ♀ 625–1364 g, [23]) breeding throughout Eurasia. There are three buzzard morphs which show marked differences in melanin coloration and many other traits including parasite load and aggression [24], [25]. It hunts its favored microtine prey over open ground and mainly breeds in small forest patches. One of its main competitors for prime nesting grounds [15], [26] is the larger and markedly more aggressive northern goshawk (♂ 517–1170 g, ♀ 820–1509 g), which opportunistically feeds on birds and mammals [27]. Goshawks have recovered from population lows in the 1960s and 1970s [21], [28], [29] and commonly breed in larger forest patches than buzzards. Both sexes of goshawk are dominant over buzzards [26], often take over buzzard territories and pose a substantial predation threat to both buzzard nestlings and adults. Predation by goshawks has previously been coarsely estimated to account for ca. 10% of the annual buzzard mortality in our study area [26], [30]. This risk is also perceived by buzzards so that breeding success, nest reuse and territory occupancy decrease with introduction of goshawk dummies and in the vicinity of goshawk nests [26], [31]. We have recorded the population dynamics and reproductive life histories of buzzards and goshawks between 1989 and 2009 in a study area densely populated by both diurnal raptors in Eastern Westphalia, Germany.

The Eagle owl is the largest owl in the world (♂ 1500–2800 g, ♀ 1750–4200 g), a broad prey and habitat generalist, and known to regularly kill other nocturnal and diurnal raptors in its territory [16]. All local raptor species, both as nestlings and as adults, may be included in its diet, causing increased risk for current and future reproduction and consequent abandonment of sites in proximity of eagle owl breeding and roosting sites [11], [12], [16]. Recolonization of a study area in Germany by eagle owls has also caused the local goshawk population to decline to one third of its previous size [32]. Among avian IG predators eagle owls have the highest fraction of consumed diurnal raptors [33] and are probably the most prevalent apex predators in avian predatory guilds overall [5]. After being exterminated through active persecution in the Federal State of Northrhine-Westphalia in 1909 [34], eagle owls reappeared in the study area in 1976 with one breeding pair. Only since the 1990s and especially after the millennium have eagle owls been recolonizing the area in greater numbers [34]. Such a slow recovery followed by rapid population growth since the 1990s has also been observed in adjacent areas such as the Federal State of Hessen [35]. Eagle owl breeding sites are restricted to a well separated ridge in the middle of our study area thus creating three plots and a natural experiment of eagle owl treatment for both mesopredators compared to control areas both to the north and south.

### Study area and setup

The study was carried out in a ca 300 km^2^ area in eastern Westphalia, Germany (8°25′ E and 52°06′ N) between 1989 and 2009. The habitat consists of pastures and meadows, interspersed with woodlots, varying between 0.001 and 7 km^2^ in size. In the southern half of the area a low mountain region reaching a height of 315 m a.s.l., the Teutoburger Wald has harboured more than two pairs of breeding eagle owls since 2003. This ridge is covered by Norway spruce *Picea abies* and beech *Fagus sylvatica*, at lower altitudes with oak *Quercus robur* and *Q. petrea* forests. To the north and south of the ridge there are plots of cultivated and urbanized landscape. In the north, forests consist mainly of beech and oak, whereas Scots pine *Pinus sylvestris* dominates in the south.

Each year we scanned all woods for active nests of diurnal raptors. We also controlled all sites in the area suitable for eagle owl breeding, mostly old quarries. A total of 355 goshawk and 1504 buzzard breeding attempts were registered that are included in the analyses. As a new territory we consider a breeding attempt located between two active or former breeding sites of the respective species, where no breeding attempt has taken place until then. As an extinct territory we consider a cluster of nesting sites, where no breeding attempt has taken place for at least 2 years and breeding has not been resumed until 2009. For each active nest we recorded coordinates with a GPS device. During the breeding season we made multiple visits to each nest to establish the approximate laying date and number of fledglings in each nest. In birds of prey the number of fledglings produced is known to correlate well with the number of recruits [36], [37], so it can serve as a surrogate of fitness. For buzzards we also recorded the identity and morph of each bird belonging to the focal pair and a coarse vole abundance score for the year (1 =  low, 2 =  medium, 3 =  high).

We consider buzzards and goshawks breeding within the central ridge (63 km^2^) to be under strong eagle owl influence, those to the south (45 km^2^) under weak eagle owl influence and those to the north (166 km^2^) under no eagle owl influence (distance between eagle owl and goshawk nests for 2004–2009 - center: mean 1.8 km, range 0.7–3.1 km; south: mean 3.3 km, range 2.5–4.1 km; north: mean 6.6 km, range 2.8–12.1 km; distance between eagle owl and buzzard nests for 2004–2009 - center: mean 1.6 km, range 0.1–3.5 km; south: mean 3.6 km, range 2.1–5.3 km; north: mean 7.3 km, range 1.3–14.7 km). Distances between nests of different species for a given year and nearest neighbor distances (NND) were estimated with the distance matrix tool of Quantum GIS 1.4.0 [38].

### Statistical analyses

Reproductive success and brood failure were analysed in generalized linear mixed models with normal and binomial error distributions respectively. Before analyses, reproductive output was standardized against mean and standard deviation of the year so that each year had a mean 0 and standard deviation 1, further termed standardized reproductive success. Territory identity was entered as random factor for goshawks and female identity for buzzards. Plot, period of eagle owl establishment (1989–2003 vs. 2004–2009), distance to the respective IG predators and NND were added as fixed factors and meaningful interactions were included in the maximum model. As the highly skewed distance to the next eagle owl could not be normalized, we decided to reduce this continuous variable into a dichotomous one: the distance between a mesopredator nest and an eagle owl nest was hence entered as a two level factor - within or more than 1.5 km. Melanin morphs of the female and male were also included in models explaining reproductive success and brood failure in buzzards as previous analyses have shown how important they can be as predictors of reproductive parameters [21], [39]. Vole score was added in models explaining buzzard breeding failure. Territory dynamics measured as the joint number of new territory establishments and extinctions in the second decade of study were analysed using χ^2^ tests. Population density was analysed in generalized linear models with normal error distribution. Plot, decade and the density of the other mesopredator entered as explanatory factors for goshawk and buzzard and vole score was added for buzzard population dynamics only. Meaningful interactions between these factors were included in the maximum models.

Model selection was based on AICc (Akaike Information Criterion corrected for small sample sizes). The relative importance of each model was estimated through ranking the models by ΔAICc =  AICc_i_-AICc_min_ (where AICc_min_ is the best model in the model subset). Model weight was estimated through the normalized Akaike weights, exp(−0.5× ΔAICc)/
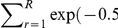
× ΔAICc_r_) and candidate models within 10% of the maximum weight are reported [40]. Statistical modelling was performed in R 2.11.1 with the packages lme4 0.999375–34 and MuMIn 0.12.2.
